# Evolution of Trefoil Factor(s): Genetic and Spatio-Temporal Expression of Trefoil Factor 2 in the Chicken (*Gallus Gallus Domesticus*)

**DOI:** 10.1371/journal.pone.0022691

**Published:** 2011-07-29

**Authors:** Zhengyu Jiang, Amy C. Lossie, Todd J. Applegate

**Affiliations:** 1 Department of Animal Sciences, Purdue University, West Lafayette, Indiana, United States of America; 2 Department of Medicine, Indiana University School of Medicine, Indianapolis, Indiana, United States of America; University of South Florida College of Medicine, United States of America

## Abstract

Trefoil factors are essential healing initiators participating in mucosal reconstitution and tissue morphogenesis, especially on the surfaces of the gastrointestinal tract. This family has been cloned and characterized predominantly from mammals and amphibians. Avian species ingest stone and grit to help digest food, which may expose their gut to severe physical conditions. To further the understanding of the function of the *TFF* gene family across species, we undertook this research to clone, sequence, and characterize the spatio-temporal expression patterns of chicken *TFF2* (*ChTFF2*) cDNA. Bioinformatics analysis of the promoter region and deduced amino acid sequence demonstrated that *ChTFF2* contained unique characteristics; specifically the chicken promoter has multiple start sites and the protein contains a series of Lys-Lys-Val repeats. Unlike mammals, where *TFF2* is detected primarily in the stomach, and occasionally in the proximal duodenum, chicken *TFF2* transcripts are found throughout the gastrointestinal tract, with major expression sites in the glandular and muscular stomach as well as evident expression in the colon, small intestine, cecal tonsil and crop. Temporal analysis of intestinal *ChTFF2* transcripts by quantitative RT-PCR showed high levels in embryos and a trend of constant expression during embryonic and post-hatch development, with a reduction occurring around hatch. Phylogenetic analysis highlighted the conservation of TFF proteins and functional divergence of trefoil domains, which suggest a transitional role in the bird during evolution.

## Introduction

The trefoil factors (TFFs) are a family of small (7–12 kDa in mammals) secretory protease-resistant peptides discovered in the 1980's [1]. These proteins have a unique trefoil-like structure, and are crucial for epithelial restitution and wound healing, especially on mucosal surfaces (reviewed by [2] [3] [4] [5] [6]). Moreover, TFFs hold tremendous therapeutic potential for preventing and treating various gastrointestinal diseases in humans [7] [8]. Named from their “three-leaf” structure, the TFF cluster molecules share a common cysteine-rich trefoil motif [2] [9]. The highly-conserved trefoil motif (also known as the P-domain) consists of the following sequence: CX_9–10_CX_9_CX_4_CCX_10_C (where C represents cysteine and X represents any other amino acid), which forms three disulfide bonds in a unique 1–5, 2–4 and 3–6 of Cys-Cys linkage structure [2]. Notably, this configuration differs from other similar protein domains such as the EGF-repeat family, which forms 1–3, 2–4 and 5–6 Cys-Cys bonds, and may allow TFFs to use distinct signaling cascades for their cellular functions, which include promoting cell migration [6].

In mammals, three members of the TFF family have been annotated: TFF1 or pS2, TFF2 or spasmolytic peptide, and TFF3 or intestinal trefoil factor (ITF) [2] [9] [10]. Structurally, TFF1 and TFF3 contain one trefoil motif with six cysteines as well as a seventh unpaired cysteine, which has been suggested to help with dimerization [2] [10]; TFF2 contains two trefoil motifs, both of which are believed to be important for proper function. *In vitro* recombinants of truncated frog TFF2 bearing one single trefoil domain lose anti-apoptotic function but still promote cell migration [11]. In addition, peptides containing four or more trefoil domains have been identified from frog stomach; these peptides are co-expressed with mucogenic cells and are proposed to possess similar functions as mammalian TFFs [12].

The genes encoding TFFs have been characterized from multiple mammals such as human, mouse, rat, dog, cat, cow, wolf, rhesus monkey, short-tailed opossum, sheep, chimpanzee and pig, as well as frog and toad [5] [9] [13] [14]. Mammalian TFFs are predominantly and profoundly expressed in the gastrointestinal tract, where the expression of each gene and peptide is delicately regulated in a tissue-specific and also topographically complementary manner [15] [16]. For example, *Tff3*, which is predominantly expressed in the lower intestine, was dramatically upregulated (40-fold) in the gastric antrum and Brunner's gland (a major site of *Tff2* and *Tff1* expression) in *Tff2*-deficient mice [17], while *Tff1*-deficient mice demonstrated increased levels of *Tff3*, and a complete loss of *Tff2*, mRNA in the stomach [18]. These findings coupled with the structural similarity among the family members, are consistent with a high degree of functional redundancy among the TFF paralogs.

However, the mechanism(s) controlling *TFF* gene expression remain unclear. The spatial localization of *TFF2* in the gastrointestinal tract varies among different species. In pigs, pTFF2 peptide has been localized to acinar cells of the pancreas [19], mucous cells of stomach and throughout the small intestine [20]. In rodents and humans, *TFF2* is abundant in gastric, pyloric and Brunner's glands, but markedly lower in small intestine and colon [3] [21]. These species-specific expression patterns highlight the potential functional diversity of *TFF* genes across species.

Most of the understanding of the function of TFFs stems from studies in rodents, humans and amphibians [12] [13]; little information is available from avian species, although a potential role for TFFs in cellular architecture assembly was recently speculated in chicken gizzard [22]. In the present study, chicken *TFF2* cDNA was cloned, sequenced and the spatio-temporal expression patterns characterized. Analysis of *TFF* genes in non-mammalian model systems provides important contributions to better understand the functional importance of the *TFF* genes in wound healing in the gut, and deepens the evolutionary understanding of the biological function of TFF proteins in animal and human health.

## Results

### 1 Cloning and analysis of the full-length ChTFF2cDNA

**Figure 1:**
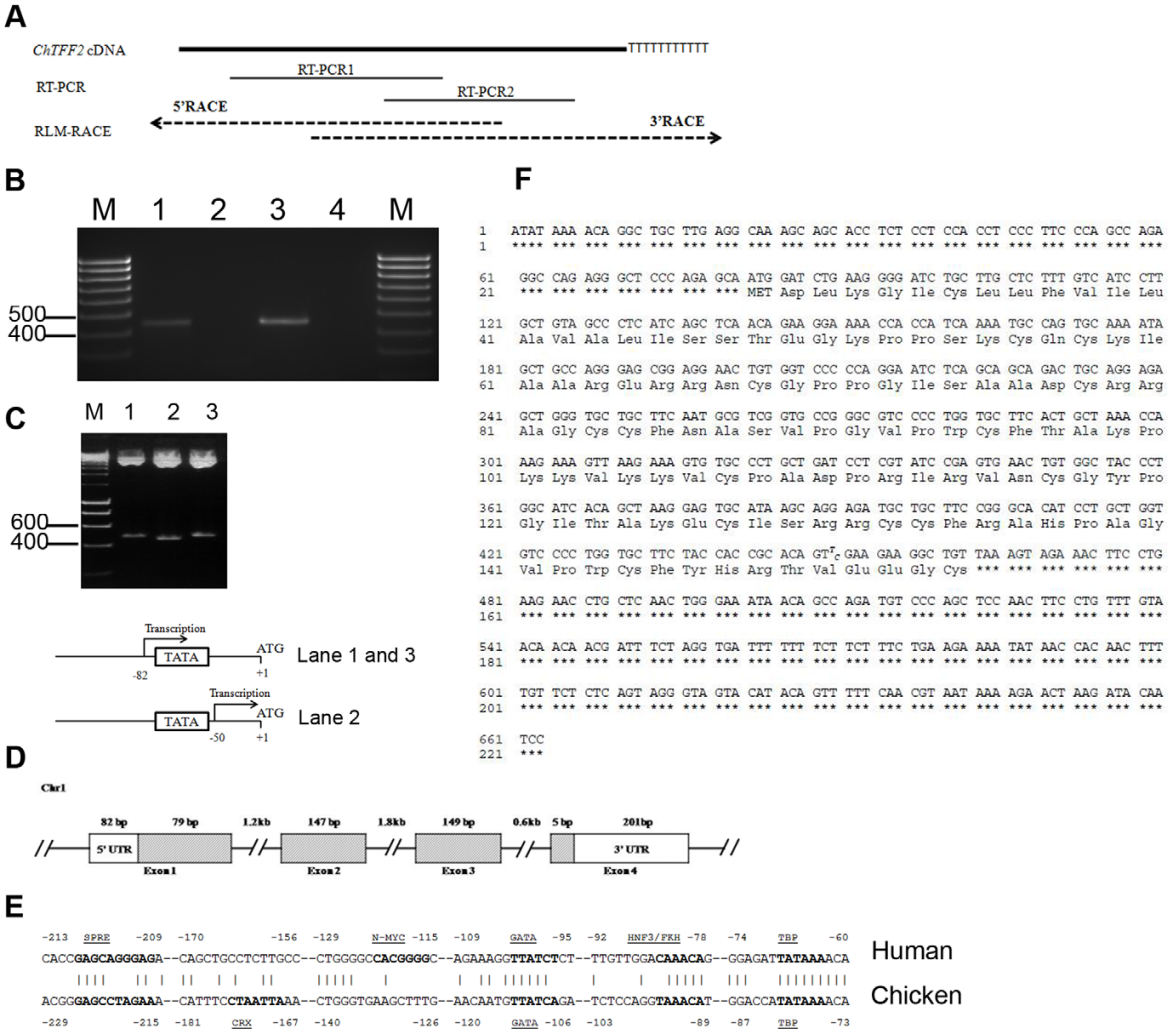
Sequence and analysis of *ChTFF2*. A: Strategy of amplying and sequencing full length *ChTFF2* cDNA. B: RLM-RACE products were electrophoresed in a 2% agarose gel. Lane 1: 3′ RACE product; lane 2: water control; lane 3: 5′ RACE product; lane 4: water control. D: Intron-exon localization of *ChTFF2* at chromosome. C: Representative electrophoresis result from *EcoRI* digestion of purified plasmids inserted with 5′ RACE product indicates alternative start site with shorter product in lane 2 as compared with lane 1 and 3; alternative transcription starting site was confirmed by sequencing determination. The ORFs are gray. E: Potential regulatory sequences in the promoter of the *ChTFF2* as compared with human; nucleotides are numbered with +1 being the A of the ATG initiation codon; target sequences for the underlined transcription factors (motif) are in bold. F: Full-length *ChTFF2* cDNA sequence and deduced amino acids.

**Figure 2:**
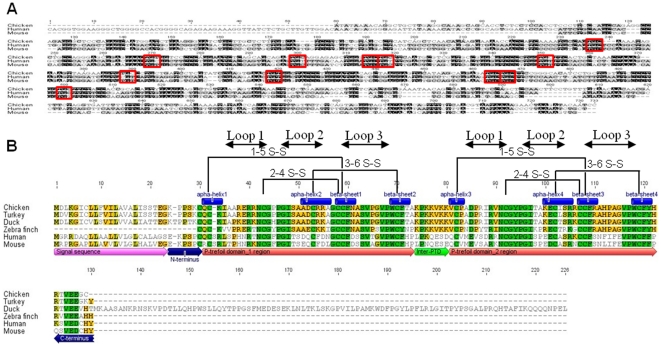
Sequence alignment. A: sequence alignments for chicken *TFF2* cDNA with human and mouse. B: Amino acid sequence alignment and analysis for chicken with predicted sequences of other avian species and human and mouse; domains are indicated below aligned sequences. PTD: trefoil domain (P domain); locations of conserved Cys bonds and secondary structural element: α-helixes, β-sheets and loops are indicated.

The *ChTFF2* cDNA spans 4.3 kb on chicken chromosome 1 (112,805,710 to 112,810,087, WUGSC 2.1/galGal3; Genome Bioinformatics Group at UC Santa Cruz [http://genome.ucsc.edu/]; (Figure 1. D)). Chicken *TFF2* shares 49.1% identity to human and mouse cDNAs (Figure 2. A). An alternative transcriptional start site (TSS) at +32 nucleotides (Figure 1. F) was identified. This resulted in the TSS mapping from upstream to 26 bp downstream of the TATA-box, which is the location of the annotated TSS in humans. A T450C single nucleotide polymorphism within the chicken cDNA was identified, implying heterogeneity within the genetic line. Alignment with the human sequence using Transcription Element Search System (TESS) indicates that possible *cis*-acting elements include TBP, GATA [23] and HNF3/FKH [24].

### 2 Deduced amino acid sequences of chicken, turkey and duck TFF2

The deduced amino acid sequence of ChTFF2 precursor predicts a 13.7-kDa, 122 residue protein that includes signal peptides, trefoil domains (TD), an inter-TD domain and terminus (Figure 2. B). Since the genomes of other avian species are less-well characterized, the deduced chicken TFF2 amino acid sequences were used in BLAST to scan the Ensembl and/or pre-Ensembl genome databases for *Meleagris gallopavo* (turkey, Ensembl), *Anas platyrhynchos* (duck, Ensembl) and *Taeniopygia guttata* (zebra finch, pre-Esembl). Alignment results showed that the degree of homology of TFF2 proteins among avian species (83% identity) is greater than that when aligned with human and mouse proteins (61.9%, Figure 2. B), and avian TFF2 is presumed to be the mammalian ortholog of TFF2.

Several amino acids are highly conserved among aves, mouse and human; The 13 Cys residues that comprise the trefoil domains and the sequences surrounding the second, fifth and sixth Cys within each trefoil domain are highly conserved [25]. Sequence comparisons indicate that the TFF2 trefoil domain consensus sequence is **C**X_6–7_RXN**C**GXPGIX_4_ **C**X_3_G**CC**FX_6_VPW**C**F. This highly conserved region suggests functional importance of these residues for trefoil factors and their structural formation, likely through disulfide bond formation, protection from protease attack, and/or receptor binding. The sequence positions of secondary structures (α-helixes and β-sheets) and loops within the ChTFF2 TD are predicted from the human sequence (Figure 2. B) [25].

### 3 Spatio-temporal expression pattern of chicken TFF2

**Figure 3:**
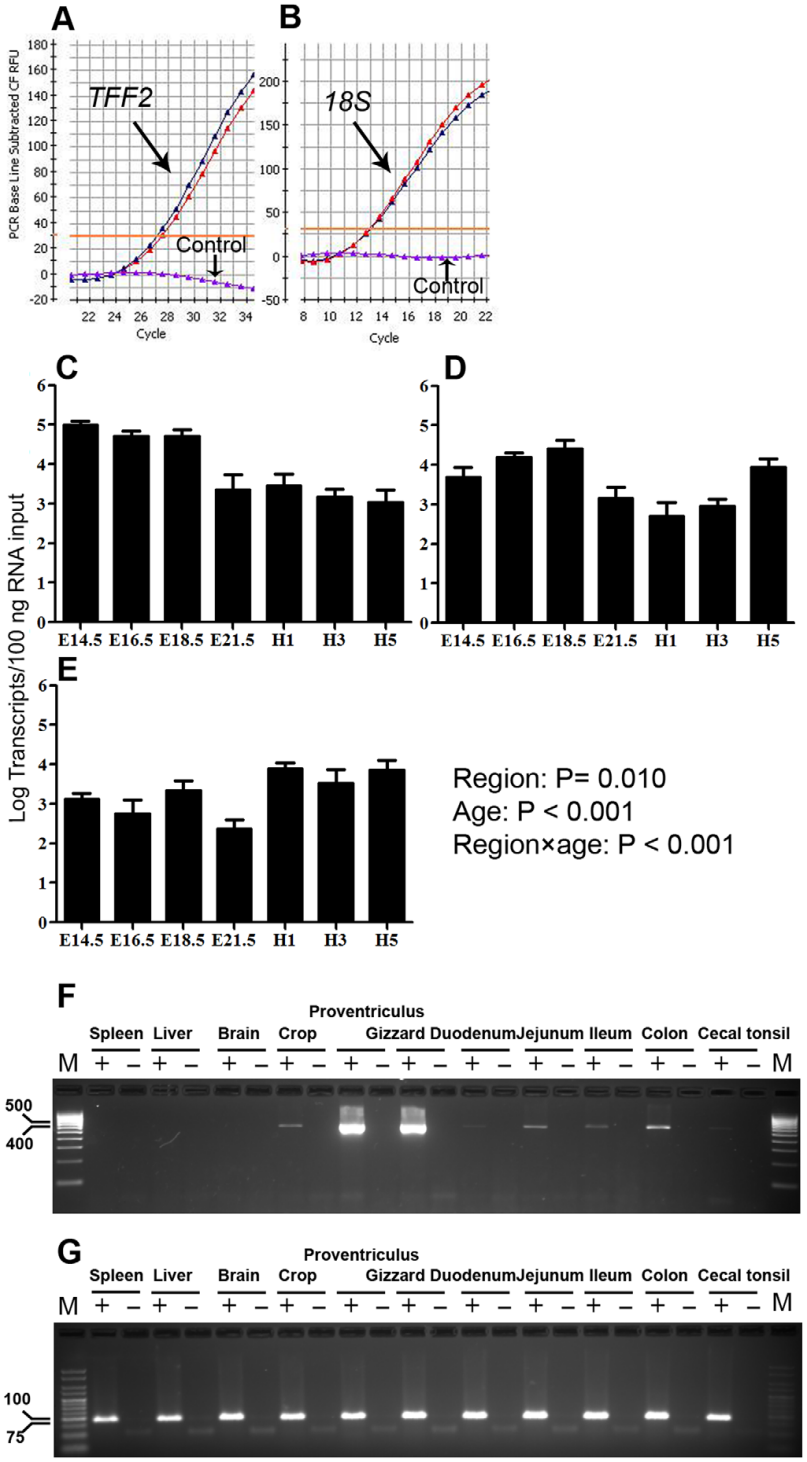
Spatio-temporal expression of *TFF2* transcripts in chicken. Representative amplification plots of quantitative PCR (duplicates) for measuring *ChTFF2* (A) and *18S* (B) in the small intestine; water was used as a control. Quantification of *ChTFF2* transcripts in embryonic (E) and post-hatch (H) ages in duodenum (C), jejunum (D) and ileum (E) using RT-qPCR; values were expressed in mean ± SE, n = 5 to 8. The expression of *ChTFF2* (F) and *18S* (G) in different tissues; forty ng of cDNA were amplified for 33 cycles using a forward primer from RT-PCR1and a reverse primer from RT-PCR2; reverse transcriptase was used (+) or omitted (−) for each tissue and all the RT-PCR products were examined by electrophoresis through a 3% agarose gel in TBE.

Distribution of *ChTFF2* mRNA was performed with tissues obtained from 3 adult laying hens using the forward RT-PCR1 primer paired with the reverse RT-PCR2 primer (Table 1, Figure 3. F and G). *ChTFF2* transcripts were detected along the gastrointestinal tract of the bird. A markedly high expression of *ChTFF2* mRNA was observed in the proventriculus (glandular stomach) and gizzard (muscular stomach). Intermediate to low levels were found in colon, crop, small intestine (duodenum, jejunum and ileum) and cecal tonsil. *ChTFF2* mRNA was not detected in spleen, liver, brain (Figure 2. F) and heart (data not shown).

**Table 1:** Primer sequences for RT-PCR, RACE and qRT-PCR.

	Forward/Reverse	Primer sequence (5′ to 3′)
*TFF2*		
RT-PCR1	Forward	CCA GAG CAA TGG ATC TGA AGG
	Reverse	CAT CTC CTG CTT ATG CAC TCC TTA
RT-PCR2	Forward	AAG TTA AGA AAG TGT GCC CTG CT
	Reverse	CCT ACT GAG AGA ACA AAA GTT GTG G
3′GSP	Forward	CCT GGT GCT TCA CTG CTA AAC CAA AG
5′ GSP	Reverse	CAG CAG GAT GTG CCC GGA AG
qRT-PCR	Forward	CTG AAC AGC AAT AAC CAC CC
	Reverse	TAA TCC CCA CAG AGA CCA CA
*18S*	Forward	GCC ACC CGA GAT TGA GCA ATA ACA
	Reverse	TAG ACA CAA GCT GAG CCA GTC AGT

Quantitative expression of *ChTFF2* mRNA in the small intestine during late embryonic and early post-hatch stages is shown (Figure 2 C, D, and E). We observed a relatively high abundance of *ChTff2* mRNA in duodenal and jejunal tissues from E14.5 to E18.5, with a slight decrease in expression from E21.5 to H1. This demarks the transition to lower post-hatch expression levels. In the ileum, *ChTFF2* mRNA levels remain relatively constant, except for a sharp reduction at hatch. Gene expression differences in the small intestine were statistically significant for age (P<0.001) and region (p<0.05). The significant region by age interaction (P<0.001) reflected a greater embryonic expression in duodenum than jejunum or ileum, with a higher post-hatch expression in ileum than duodenum and jejunum.

### 4 Phylogenetic tree of TFF2

**Figure 4:**
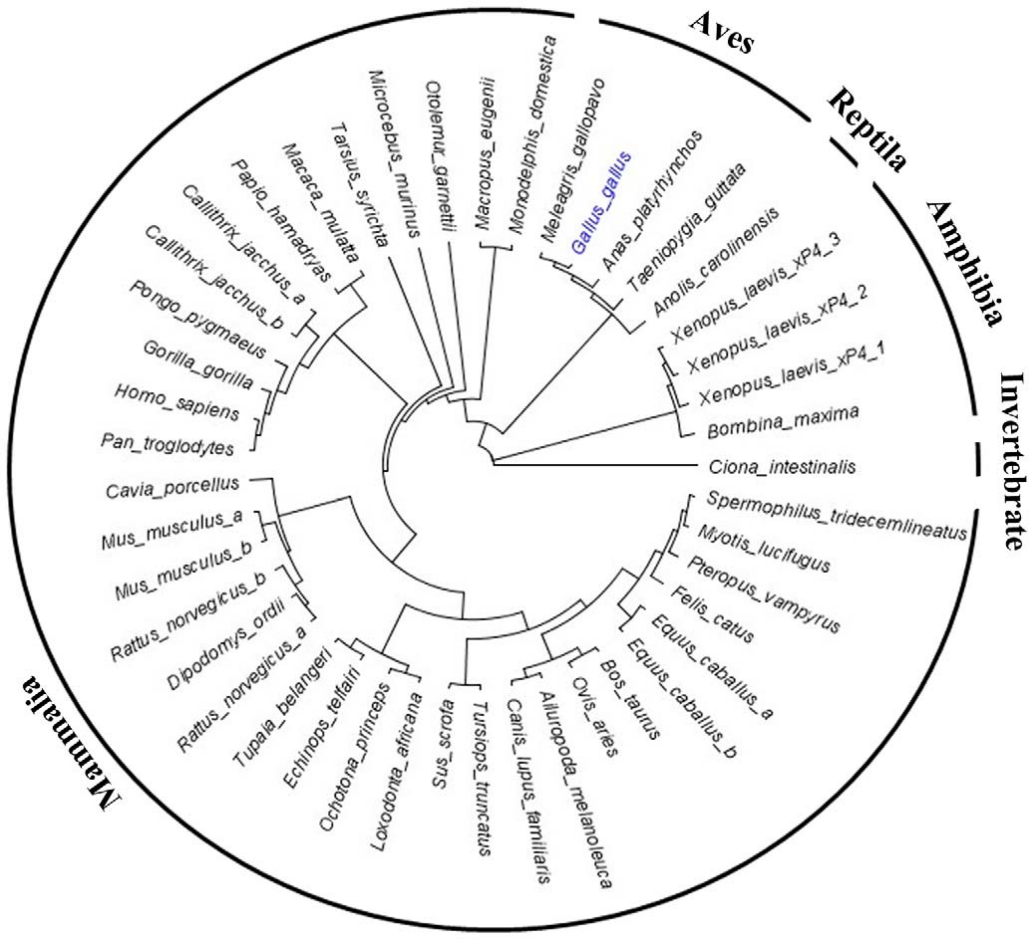
Phylogenetic tree of TFF2. Forty five TFF2 proteins and related structure were aligned by using ClustalW, and the neighbor-joining tree derived by the same program was shown. *A. carolinensis*: anole lizard. *G.gallus*: chicken. *P. hamadryas*: baboon. *A. melanoleuca*: giant panda. *G. gorilla*: gorilla. *P. pygmaeus*: orangutan. *A. platyrhynchos*: duck. *H. sapiens*: human. *P. troglodytes*: chimpanzee. *B. maxima*: toad. *L. africana*: elephant. *P. vampyrus*: megabat. *B. Taurus*: cow. *M. domestica*: opossum. *R. norvegicus*: rat. *C. intestinalis*: vase tunicate. *M. eugenii*: wallaby. *S. scrofa*: pig. *C. jacchus*: common marmoset. *M. gallopavo*: turkey. *S. tridecemlineatus*: squirrel. *C. lupus familiaris*: dog. *M. lucifugus*: microbat. *T. belangeri*: tree shrew. *C. porcellus*: guinea pig. *M. mulatta*: macaque. *T. guttata*: zebra finch. *D. ordii*: kangaroo rat. *M. murinus*: mouse. *T. syrichta*: tarsier. *E. caballus*: horse. *O. aries*: sheep. *T. truncatus*: dolphin. *E. telfairi*: lesser hedgehog tenrec. *O. garnettii*: bushbaby. *X. laevis*: African clawed frog. *F. catus*: cat. *O. princeps*: pika.

**Figure 5:**
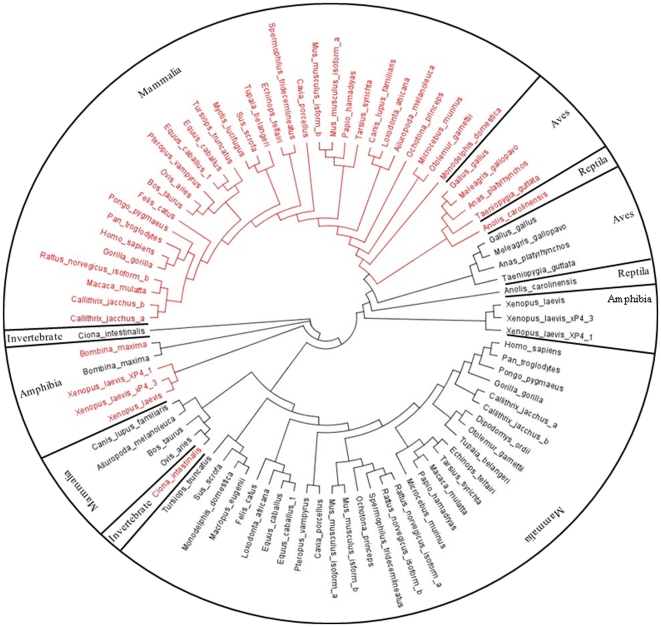
Phylogenetic tree of trefoil domains of TFF2. TD1 (red) and TD2 (black) were extracted from a range of animals as listed in Figure 4, aligned by using ClustalW, and analyzed with the neighbor-joining method.

To explore the evolutionary relationship of chicken TFF2 homologues, phylogenetic analyses of TFF2 was performed in a variety of vertebrate and invertebrate species (Figure 4, see Figure S1 for information on distance and sequence alignment). Protein sequences were retrieved from the NCBI, Ensembl and pre-ensembl databases. Since the genomes of some species were poorly characterized, their TFF2 homologues were obtained by genome-wide scans with known TFF2 proteins from closely related species. To further investigate the functional divergence of TFF2, functional domains of TFF2 sequences were phylogenetically analyzed separately. All two trefoil domain (TD) extracted from TFF2 proteins were used in the phylogenetic studies and gave rise to a tree similar in topology for each TFF2 TD to that of TFF2 in Figure 4 (Figure 5).

## Discussion

### 1 Sequence analysis and structure prediction of ChTFF2

Results confirm that computational annotation correctly predicted the putative chicken *TFF2* nucleotide sequence, with the exception of identifying additional 3′ and 5′ UTR sequences. Despite scanning the chicken genome and other chicken cDNA clones with TFF sequences from the Pfam trefoil motif in human, mouse and other species with Genewise (http://www.ebi.ac.uk/Tools/Wise2/), no other chicken *TFF* candidate genes were detected. However, in mammals, there are three TFF members. All three genes map to a single chromosome and reside near each other in the following tandem orientation: 5′-TFF1-TFF2-TFF3-3′. It has been suggested that this region is a consequence of gene duplication and exon-shuffling events during evolution [26] [27].

The *TFF2* promoter contains a complex enhancer region. Comparison of the chicken *TFF2* promoter sequences with the human homologue revealed several conserved regions (Figure. 1 E). Previous studies of the human *TFF2* promoters demonstrated that the HNF3 *cis*-acting element is located between −10 and −16 upstream of the TATAA box that is accessed during the acute-phase response of TFF2 peptide [24]. Here, we document a similar HNF3 binding motif (TAAACAT) within the chicken gene. The predicted GATA enhancer element also is conserved between chicken and human. However, the presence of the specific SPRE *cis*-acting element that has been reported to be responsible for TFF2 autoinduction [28] in chicken is uncertain based on promoter sequence alignment.

The fact that trefoil loops are highly conserved during evolution, particularly the amino acids surrounding the Cys residues, indicates that these structures are important for the functionality of this protein family. Comparison of human trefoil factors via H nuclear magnetic resonance (NMR) showed distinct structural and electrostatic properties of the loop2 and loop3 regions, and as the authors suggested, it is possible that the functional diversity can be increased by the interactions of each loop with different substrates [25]. In aves, the trefoil motifs are highly conserved, with 8 to 9 residues more of intraspecific consensus compared to mammals. This unique preserved region among avian species may indicate species-specific structural or functional characteristics. For example, the avian inter-TD domain is rich in negatively charged Lys, and contains Lys-Lys-Val repeats that are not present in mouse or human. This Lys-Lys-Val structure, which lies near secondary structure elements, could protect these regions from proteolytic processing [29].

Even though careful scrutiny of the available genome resources failed to detect multiple *TFF* genes in chicken, it is possible that other chicken *TFF(s)* exist in gaps that have yet to be sequenced or in unmapped regions of the genome. Also, it is possible that the avian counterpart(s) may have fewer member(s) or additional or even distinct functional domains that differ significantly from their mammalian counterparts. For example, *in vitro* studies in the toad (*Bombina maxima*) have shown a 2-fold cell motility-inducing ability at 100-fold lower concentration than that of human TFF2 [11]. Despite the fact that *TFF* are highly conserved among species, it is probable that non-mammalian homologues may have additional or even distinct functional characteristics from mammalian TFF proteins.

### 2 Spatio-temporal ChTFF2 transcription – different expression pattern from mammals

RT-PCR results demonstrated that the major sites of *ChTFF2* expression are located within the glandular and muscular stomach, which is equivalent to the expression pattern observed in the mammalian stomach [30] [21]. The colon, as well as small intestine and crop, show intermediate levels of *ChTFF2*. However, both mice and humans lack detectable *TFF2* expression in the small intestine at both the mRNA and protein levels, whereas *TFF3* is observed at relative high abundance in the small intestine [2] [3]. In rodents, the gastric antrum and Brunner's gland, but not the lower intestine, are the two major sites of *Tff2* expression under physiological conditions [17]. However, a relatively lower level *ChTFF2* detected in duodenum is likely because avian species lack the Brunner's gland in the duodenum [31]. The *ChTFF2* expression pattern may be similar to pigs, where immunoactive pTFF2 were detected throughout the intestine [20]. However, the duodenal *ChTFF2* mRNA transcripts detected at embryonic day 14 are 1 to 2 logs higher than other segments of the small intestine.

In the mouse, *TFFs* are detected by embryonic day 13 [32]. During this stage, patterning and organogenesis has already occurred. Organs at this stage may require protective mechanisms similar to those necessary in adulthood. Indeed, upregulation of *Tff* was observed in the embryological gut following physical wounding, suggesting a healing role of *Tff* prior to birth. In addition, recent reports showed that human milk is rich in intact TFF. These studies propose that *TFFs* are associated with the development of the fetal gut and protection against various diseases [33].

Unlike mammals, which receive bioactive peptides, including TFF, from a maternal supply, avian species may require significantly higher expression levels and storage of TFF(s) during embryonic stages for adequate embryonic gut development and post-hatch protection of the gut against potential stressors. The present study examined the expression pattern of *ChTFF2* transcripts during late embryogenesis and early after hatching by quantitative RT-PCR. The small intestine was a primary focus because in chickens, *TFF2* is expressed in the small intestine at a relatively high level; in humans and rodents, *TFF2* is normally absent in the small intestine, indicating that there may be different functional properties of TFF2 in chickens compared to mammals. Whole mount *in situ* hybridization studies demonstrated that the mouse *Tff2* gene was confined to the stomach and Brunner's glands during embryonic development, with stochastic expression detected in predominate small intestine and caecum at embryonic day 17.5 [32]. qRT-PCR date from this study reveal a punctuate increasing pattern of *TFF2* mRNA expression following embryonic and post-hatch development. This differs from developmental expression of rat *Tff3*, the intestinal trefoil factor, where the expression of *TFF3* commences late in gestation and increases post-natally until adulthood [34]. In the chicken, relatively higher expression of *ChTFF2* in the duodenum is greater in embryos than after hatch; whereas the ileum (distal small intestine) is higher post-hatch. This expression shift suggests a spatial transition of expression abundance from upper intestine to lower intestine as the gut matures.

### 3 Role of aves in phylogeny of TFF2

The phylogenetic analysis helps to understand the role of TFF2 homologues in the conservation and divergence among different animal species, particularly the transitional position of chicken TFF2 during the evolutionary timeline. Avian TFF2 orthologs are phylogenetically closer to reptiles than other species, and their divergence occurred before mammals. This relationship is even more evident when analyzing functional domains (TD). Interestingly, the similarity among functional TD domains is greater within avian species, which share a uniform relatively high TD identity (50%) than that from most of other species. Inferred neighbor-joining tree of TFF2 domains suggests that the two functional domains (TD1 and TD2) are not evolutionarily distinct and may be generated by domain duplication.

To further investigate whether amino acid substitutions between species and functional domains could have caused functional diversification, Type-1 functional divergence between species clusters and TD domain clusters were estimated by posterior analysis using the DIVERGE program algorithms [35] [36] (Table 2). This allows evaluation of the shifted evolutionary rate at each sequence position and defines important amino acid residues responsible for altered functional constraints. Significant site-specific altered selective constraints between different animal species were observed (*θ*>0), especially between aves and amphibians (N = 78 when cut-off value is 0.67). The coefficient of functional divergence between TD1 and TD2 as well as the predicted number of divergent amino acid residues are greater in aves versus mammals, suggesting a higher degree of altered functional divergence between the two functional domains. This is consisted with the hypothesis that there is a higher likelihood that each domain of TFF2 confers a specific protein function.

**Table 2:** Functional divergence estimated in TFF2 gene and domains.

Comparison	Sites^1^	θ^2^	SE^3^	LRT^4^	N(0.5)^5^	N(0.67)^5^
TFF2						
Mammal *vs.* Aves	78	0.25	0.22	0.23	2	1
Mammal *vs.* Amphibian	78	0.67	0.41	0.67	77	28
Aves *vs.* Amphibian	78	0.87	0.45	0.85	78	78
TD1 *vs.*TD2						
Mammal	29	0.40	0.18	10.61	6	4
Aves	42	0.59	0.36	5.30	36	12

1 Sites: the total number of amino acid residues.

2 θ is the coefficient of type I functional divergence; θ>0 suggests altered functional constrains occurred.

3 SE: standard error.

4 LRT is a likelihood ratio test.

5 N(0.5) and N(0.6) means the numbers of divergent residues when the cut-off value is 0.5 and 0.67, respectively.

Overall, this study characterized encoding sequence and spato-temporal expression patterns of the chicken TFF2, and analyzed deduced amino acid sequences, structures and sequence homologies among different species.. Knowledge of sequence, phylogenic and expression information will allow further experiments to investigate its functionality and potential implications for wound healing in the gastrointestinal tract.

## Materials and Methods

### Tissue sampling

All procedures and protocols were approved by Purdue University Animal Care and Use Committee. Fertile chicken eggs (n = 720) were obtained and incubated (Jamesway Incubator Company Inc., Cambridge, Ontario, Canada). Since intestinal segments can be identified by embryonic day 14,5, embryonic intestinal tracts were dissected (from day 14.5 to days 16.5, 18.5, and E21.5) and on post-hatch chickens (days 1, 3, 5). At each stage, 5 to 8 animals per age group were assessed. We dissected the following intestinal regions: duodenum (from the ventriculus to the end of the pancreatic loop), jejunum (from the duodenum to the yolk sac), and ileum (from the jejunum to the ileal-cecal junction). Each segment was placed in RNALater (Ambion Inc., Austin, TX) and snap-frozen.

To examine temporal expression of *ChTFF2* RNA transcripts, approximately 50 mg of the mid-length of duodenum, jejunum and ileum were used for RNA isolation. 35 week-old adult White Leghorn hens were euthanized by over-dose of carbon dioxide. Tissue samples were collected and immediately frozen in liquid nitrogen for subsequent RNA isolation.

### RNA isolation and reverse transcription

RNA isolation was performed using TRIzol® reagent (Initrogen Inc., Carlsbad, CA) followed by RNA purification with the DNA-*free™* DNase Treatment and Removal Kit (Ambion Inc., Austin, TX). Purified RNA was quantified by Nanodrop and aliquoted. RNA integrity was verified by electrophoresis on a 1% agarose gel. For RT-PCR, complementary DNA (cDNA) was synthesized and diluted as described [37].

### Full length cDNA amplification with RNA-ligase-mediated rapid amplification of cDNA ends (RLM-RACE)

RLM-RACE was performed using the GeneRacer™ RLM-RACE kit (Invitrogen Inc., Carlsbad, CA) according to the manufacturer's instructions. Briefly, full-length capped mRNA was obtained by treating purified total RNA with calf intestinal phosphatase (CIP), which removes fragmented mRNA and non-mRNA. The protective 5′ cap structure from full-length mRNA was then dephosphorylated with tobacco acid pyrophosphatase (TAP) to allow subsequent ligation of an RNA oligo to the 5′ end by T4 RNA ligase. Ligated mRNA was reverse transcribed using SuperScript™ III RT and GeneRacer™ Oligo dT primers. To obtain the 3′ end of the *ChTFF2* transcript, first strand cDNA was amplified with Advantage© 2 system (Clontech Laboratories, Inc., Mountain View, CA) using the 3′ anchor GeneRacer™ primer and a forward gene specific 3′ primer (GSP). To amplify the 5′ end of *ChTFF2*, a reverse complement 5′-GSP was used with the anchor 5′ GeneRacer™ primer. GSPs were designed from initial resultant sequencing contig of cDNA amplicons produced by two internal primer pairs (Figure 1. A).

### Cloning and sequencing

All products were cloned into the pCR-4 TOPO vector and chemically transformed into TOP10 *E. coli* cells (Invitrogen Inc., Carlsbad, CA). Ten to twenty clones from each transformation were grown in LB broth overnight. Plasmids from each clone were prepared and purified using a Quicklyse Miniprep kit (Qiagen Inc., Valencia, CA) and digested with *EcoRI*. Digested fragments were resolved by gel electrophoresis on 1.5% agarose, 0.5× TBE gels. Three separate clones were sequenced bidirectionally using Big Dye 3.1 (ABI, Life Technologies). The resultant sequences were aligned using Sequencher™ Software (Gene Codes Corp., Ann Arbor, MI).

### Sequence analysis and phylogenetic tree

The full length chicken *TFF2* coding sequence was translated using an online Open Reading Frame Finder (http://www.ncbi.nlm.nih.gov/gorf/gorf.html). The amino acid sequence was compared to sequences from other species available in GenBank (http://www.ncbi.nlm.nih.gov/genbank/), Ensembl (http://pre.ensembl.org/index.html) and Pre Ensembl (http://uswest.ensembl.org/index.html). All the sequences were aligned using ClustalW (http://www.ebi.ac.uk/Tools/msa/clustalw2/). A phylogenetic tree was constructed using neighbor-joining method based on sequence distance matrix, and displayed using Geneious™ Software (Biomatters Ltd, Auckland, New Zealand). The search for potential transcriptional binding sites was performed using an online Transcription Element Search System (TESS, http://www.cbil.upenn.edu/cgi-bin/tess/tess). Published human *cis*-acting elements for *TFF2* were compared as a reference.

### Quantitative RT-PCR

Primer pairs for qRT-PCR analysis were optimized, and PCR products were cloned (into the pCR-4TOPO vector) and sequenced for confirmation. Assays were carried out in 15 µL reactions using iQ SYBR Green Supermix (Bio-Rad Laboratory, Hercules, CA) with diluted first-strand cDNA. qRT-PCR programs for *ChTFF2* and *18S RNA* were designed as: 5 min at 95°C, 40 cycles of 95°C for 15 sec, 56°C or 57°C for 15 sec, 72°C for 15 sec and 82°C or 83°C for 15 sec data collection, followed by 80 cycles for melting curve analysis. All cDNA samples calculated from 100 ng of total RNA per reaction were assayed in duplicate. Quantification standards were comprised of four 100-fold dilutions of purified plasmid DNA (10^8^ to 10^2^ or 10^7^ to 10^1^ molecules) and assayed in triplicate in each plate with R square values of 0.99 or above. Standards were used to calculate a linear regression model for threshold cycle (Ct) relative to transcript abundance in each sample. The log value of *ChTFF2* transcript starting abundance was calculated from the Ct values corrected by a factor calculated from *18S RNA* as previously described [37].

### Statistics for quantitative PCR

Log values for transcript abundance from each sample duplicate were subjected to ANOVA using the MIXED procedure of SAS for intestinal region and age effects, as well as the region × age interaction. Random effect was defined as animal nested within region × age. An alpha of 0.05 was used for all PCR analysis.

## Supporting Information

Figure S1 **Evolutionary tree and sequence alignment of TFF2.**
(TIF)
