# Supplementary figures and images for: Evolution of Trefoil Factor(s): Genetic and Spatio-Temporal Expression of Trefoil Factor 2 in the Chicken (Gallus Gallus Domesticus)
Source: PLoS One. 2011 Jul 29;6(7):e22691. doi: 10.1371/journal.pone.0022691 (PMC3146476; doi:10.1371/journal.pone.0022691)

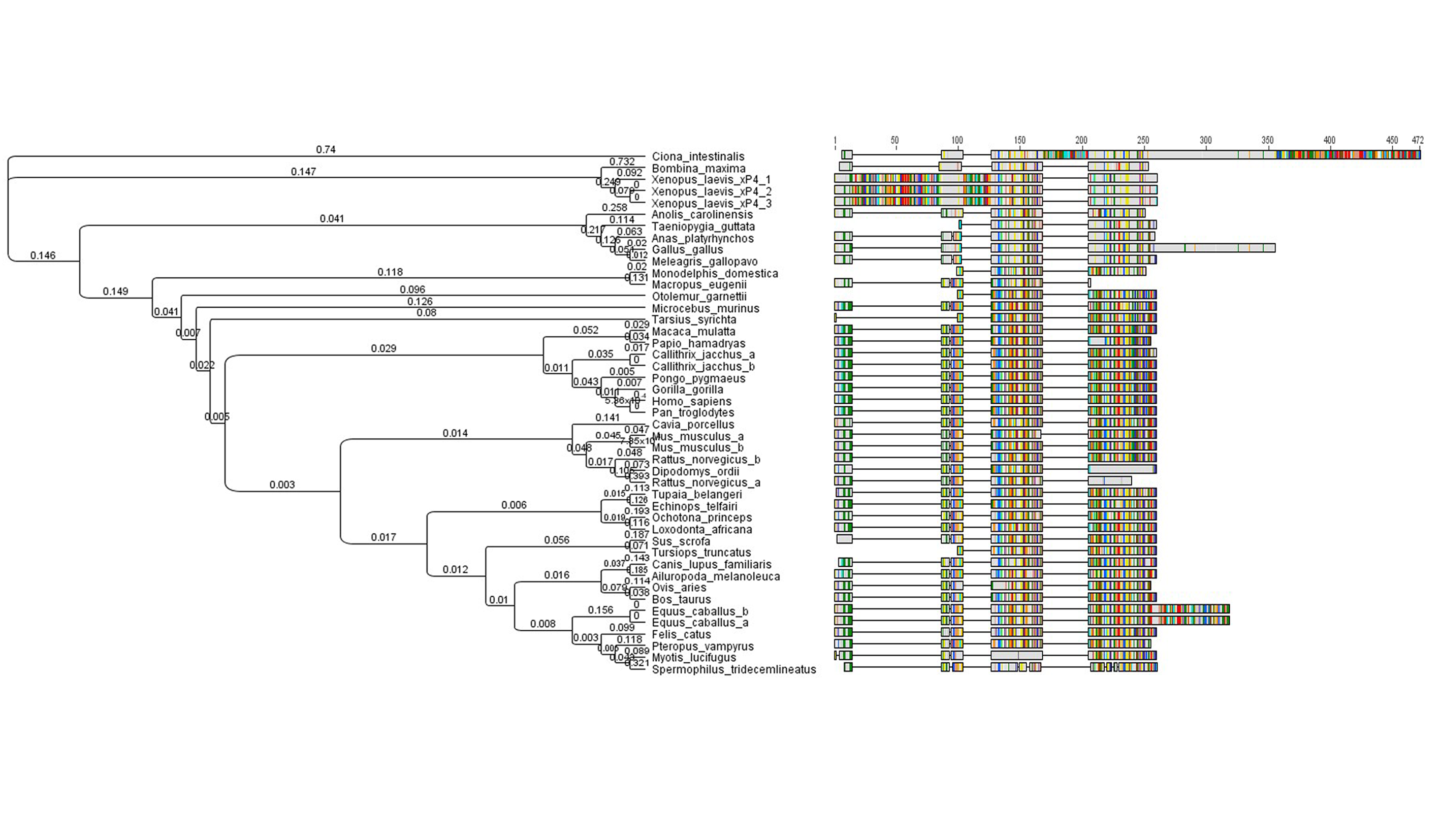

Supplement: Figure S1 — Evolutionary tree and sequence alignment of TFF2. (TIF) [file pone.0022691.s001.tif]
